# Institutional Questions

**Published:** 1900-06-30

**Authors:** 


### INSTITUTIONAL QUESTIONS.

**Patients' Payments.**

"Londoner" writes: Will you kindly tell me on what terms paying patients are admitted to the Great Northern Central Hospital, and if the accommodation is in a general ward or in separate rooms?

*** There are small separate wards for paying patients at the Great Northern Central Hospital, and the maximum charge is £2 2s. per week.

**The Deaths from Enteric.**

"A Nurse" asks: What is the reason of the appalling lists of deaths from enteric daily reported in the papers from Bloemfontein and other places in South Africa? Is the type of disease peculiarly virulent, or is the fault to be found in the care meted out to the men when ill? Very little notice seems to be taken of these terribly long lists appearing day after day, yet it is clear that the men are dying by hundreds every week. Are the filtering arrangements quite useless, or is it impossible to prevent the men from drinking contaminated water?

*** We have at present no statistics showing what is the proportion of deaths to cases among those suffering from enteric fever in South Africa. As regards the filtering arrangements, it must be remembered that there is no really reliable extempore means of purifying water other than boiling.

**Association of Asylum Workers.**

A correspondent writes: Will you give me the address of the head-quarters of the Association of Asylum Workers? If you could also give me some particulars respecting this society, its rules and objects, and terms of membership, I should be glad.

*** The Association of Asylum Workers was founded in 1895, largely through the exertions of Miss Honnor Morten, for the following objects: (1) To band together workers in asylums for their mutual benefit; (2) to educate the public to better understand the difficulties of asylum work, and to give greater respect and more consideration to those engaged in the care of the insane; (3) to circulate a monthly journal called *Asylum News* as one means of obtaining the above objects; (4) to provide a "Home of Rest and Nursing" for those engaged in asylum work, no such home being in existence. There are three classes of members—life members, associate members, and ordinary members. Sir James Crichton Browne is president of the association, and Dr. G. E. Shuttleworth, Ancaster House, Richmond Hill, hon. secretary and editor of *Asylum News*. An asylum workers' employment bureau has been established at 10, Thayer Street, Manchester Square.

